# Fatty acid binding protein 4 enhances prostate cancer progression by upregulating matrix metalloproteinases and stromal cell cytokine production

**DOI:** 10.18632/oncotarget.22908

**Published:** 2017-12-04

**Authors:** Mingguo Huang, Shintaro Narita, Takamitsu Inoue, Atsushi Koizumi, Mitsuru Saito, Hiroshi Tsuruta, Kazuyuki Numakura, Shigeru Satoh, Hiroshi Nanjo, Takehiko Sasaki, Tomonori Habuchi

**Affiliations:** ^1^ Department of Urology, Akita University Graduate School of Medicine, Akita 010-8543, Japan; ^2^ Research Center for Biosignal, Akita University Graduate School of Medicine, Akita 010-8543, Japan; ^3^ AMED-CREST, Japan Agency for Medical Research and Development (AMED), Tokyo 102-0004, Japan; ^4^ Department of Clinical Pathology, Akita University Graduate School of Medicine, Akita 010-8543, Japan

**Keywords:** fatty acid binding protein 4, cancer microenvironment, high-fat diet, prostate stromal cell, prostate cancer

## Abstract

Fatty acid binding protein 4 (FABP4) is an abundant protein in adipocytes, and its production is influenced by high-fat diet (HFD) or obesity. The prostate stromal microenvironment induces proinflammatory cytokine production, which is key for the development and progression of prostate cancer (PCa). Here, we show that high FABP4 expression and its secretion by PCa cells directly stimulated PCa cell invasiveness by upregulating matrix metalloproteinases through phosphatidylinositol 3-kinase and mitogen-activated protein kinase signaling pathways. In addition, prostate stromal cells augmented PCa cell invasiveness by secreting interleukin-8 and -6 in response to FABP4. This was abrogated by the FABP4 specific inhibitor, BMS309403. Furthermore, a mouse xenograft experiment showed HFD enhanced PCa metastasis and invasiveness by the upregulation of FABP4 and interleukin-8. Clinically, the serum level of FABP4 was significantly associated with an aggressive type of PCa rather than obesity. Taken together, FABP4 may enhance PCa progression and invasiveness by upregulating matrix metalloproteinases and cytokine production in the PCa stromal microenvironment, especially under HFD or obesity.

## INTRODUCTION

Prostate cancer (PCa) is one of the most commonly diagnosed and leading causes of cancer-related death in Western countries and Japan [1-3]. Many epidemiological studies suggest that the risk of PCa is increasingly associated with environmental factors such as high-fat diet (HFD) and obesity [4-6]. HFD or obesity may affect gene expression, cellular activity, and other important changes in circulating biological factors related to PCa aggressiveness [7-11]. The prostate stromal microenvironment has emerged as a key factor in the growth and development of PCa [12]. Prostate stromal fibroblasts are a common component of the prostate stroma, and crosstalk with adjacent cancer cells might influence tumor progression by the secretion of proinflammatory cytokines and growth factors [13, 14]. Recent studies have shown that periprostatic inflammation induced by HFD and obesity was strongly associated with the progression of various cancers including PCa [15, 16].

Fatty acid binding protein 4 (FABP4) is an abundant protein in mature adipocytes and adipose tissue, and its expression and production are influenced by HFD or dietary components [17-19]. FABP4 may have an important role in the restive prostatic stroma and influence PCa progression, especially under obesity and/or HFD conditions [20]. However, the expression of FABP4 in the prostate stroma and it role in PCa is poorly understood. FABP4 is a cytosolic 14–15 kDa protein that affects lipid flux, and its expression is strongly upregulated during adipocyte differentiation [21-23]. Recent studies showed that FABP4 translocates to the nucleus where it interacts with peroxisome proliferator-activated receptor γ (PPAR γ) to affect the growth and differentiation of cells, including normal prostate cells and PCa cells [24-26]. In addition, FABP4 binds to a variety of hydrophobic ligands, such as long chain fatty acids, retinoids and different vitamins, and these dietary components represent a feedback loop that regulates FABP4 expression [27]. Interestingly, phosphatase and tensin homolog deleted on chromosome 10 (PTEN), a significant tumor suppressor of various cancers including PCa, interacts with FABP4 [28], and *FABP4* expression was higher in Pten-null keratinocytes compared with normal cells [29].

Clinical studies reported FABP4 in the cultured medium of adipocytes had physiological activity, and that circulating levels of FABP4 were strongly linked with obesity and metabolic diseases [17-19], and had a significant role in type 2 diabetes and atherosclerosis by acting on metabolic and inflammatory pathways [30, 31]. The elevated expression of FABP4 was reported in various types of cancer cells, and in cancer angiogenesis and metastatic proliferation in ovarian cancer, non-small cell lung cancer and breast cancer [32-34]. Furthermore, FABP4 promoted ovarian cancer metastasis via the direct transfer of lipids from adipocytes to invasive cancer cells to provide fatty acids for rapid tumor growth [35]. FABP4 was highly expressed in skeletal metastatic PCa in a mouse model and was involved in human PCa bone metastasis [20]. Therefore, exogenous FABP4 might be involved in human PCa progression by activating the phosphatidylinositol 3-kinase and mitogen-activated protein kinase (PI3K) pathway independent from its binding to fatty acids [36].

The aim of this study was to delineate the role of FABP4 in the progression of PCa with a focus on its interaction with the stromal cell tumor microenvironment. We investigated the expression and role of FABP4 in PCa cells and prostate stromal cells. Second, we investigated whether FABP4 enhances the invasive capacity of PCa cells directly or indirectly *via* stromal cells. Furthermore, we examined the mechanisms involved in the direct or indirect enhancement of cancer cell invasive capacity. We also investigated whether FABP4 is involved in the PCa progression enhanced by a high fat diet (HFD) using a mouse xenograft model. Finally, of clinical relevance, we examined whether serum FABP4 levels correlated with the clinicopathological parameters in patients with PCa.

## RESULTS

### Secreted FABP4 promotes PCa invasiveness by stimulating prostate stromal cells to secrete proinflammatory cytokines

**Figure 1:**
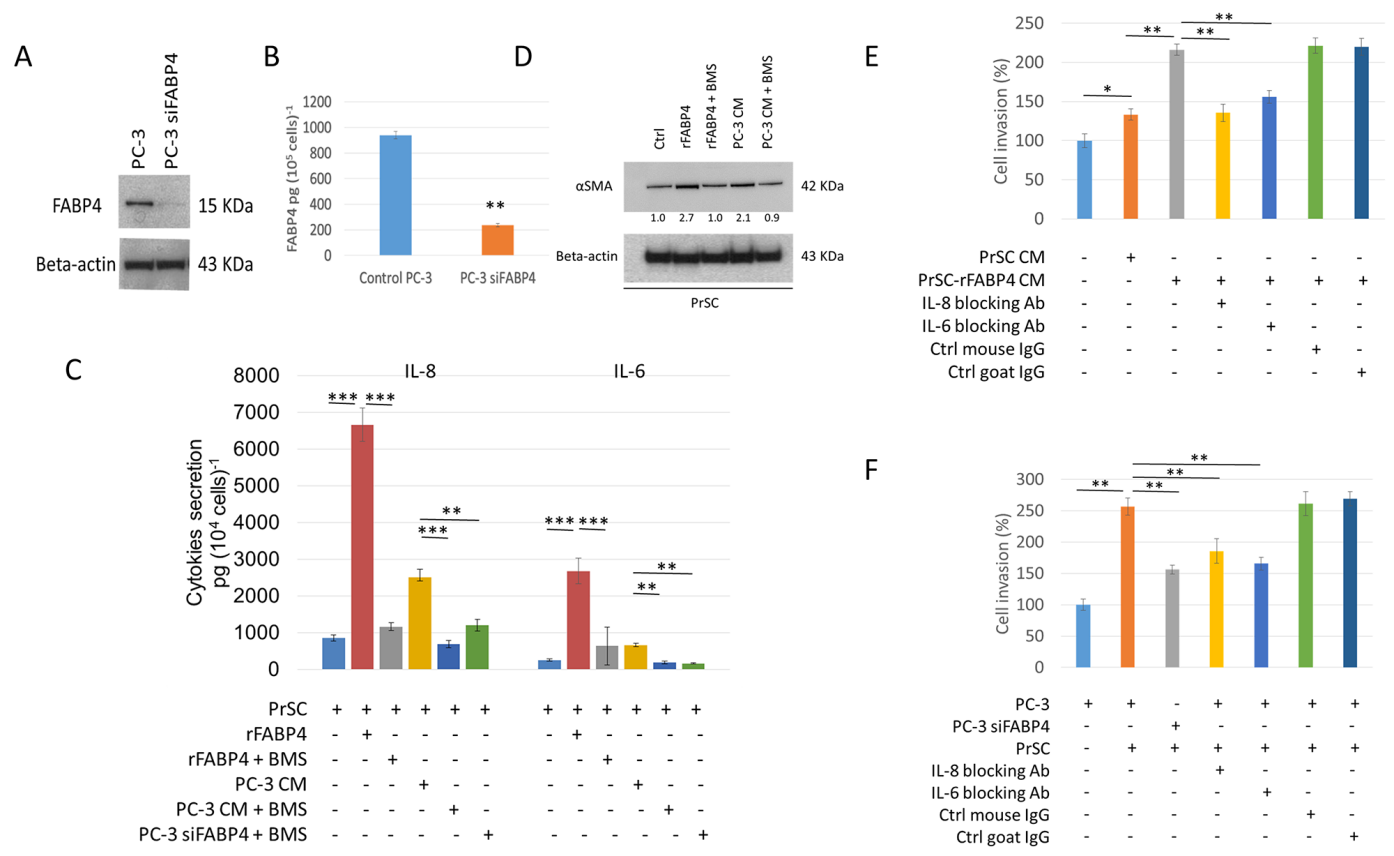
Expression and secretion of FABP4 promotes PCa invasiveness by activating PrSC to produce proinflammatory cytokines **(A** and **B)** Expression and secretion of FABP4 in PCa cells. Overall, 1 × 10^5^ PC-3 cells were cultured in a 35-mm dish, and treated with or without 50 nM FABP4 siRNA-1 for 24 hours. Equal amounts of proteins (10 μg) from the cultured cells were subjected to anti-FABP4 and anti-beta-actin staining (A). The mean FABP4 level in the conditioned medium (CM) was significantly lower in PC-3 cells treated with FABP4 siRNA-1 (235.4 ± 11.6 pg per 10^5^ cells) compared with control PC-3 cells (938.8 ± 29.0 pg per 10^5^ cells) (*P* = 0.007, B). ^**^P < 0.01. **(C)** Cytokine secretion of PrSC cells stimulated with FABP4. Overall, 2 × 10^4^ PrSC cells were treated with or without 100 ng ml^-1^ rFABP4 or PC-3 conditioned medium (PC-3 CM) obtained from (A), in the presence or absence of 30 μM BMS309403 (BMS) for 24 hours. IL-8 and IL-6 levels were significantly higher in PrSC treated with rFABP4 or PC-3 CM (6662.0 ± 457.1 and 2678.1 ± 342.4 pg (10^4^ cells)^-1^, *P* = 0.0003 and *P* = 0.0002; and 2506.1 ± 218.7 and 654.8 ± 51.0 pg (10^4^ cells)^-1^, *P* = 0.0044 and *P* = 0.021; respectively), and the effect was markedly inhibited in the presence of BMS309403. In addition, IL-8 and IL-6 levels were significantly lower in the conditioned medium of PrSC cultured in PC-3 CM treated with FABP4 siRNA-1 compared with untreated PC-3 CM. Mean ± S.D., ^**^P < 0.01, ^***^P < 0.001. **(D)** Western blotting using anti-αSMA and anti-beta-actin antibodies of PrSC proteins treated with various agents described in (C). **(E** and **F)** PrSC augmented PC-3 cell invasiveness by secreting IL-8 and IL-6 in response to FABP4 secreted by PC-3 cells. (E) The relative value (%) of *in vitro* Matrigel invasion assay under each conditioned media is shown based on PC-3 cells with PrSC CM. PrSC-rFABP4 CM: conditioned media of PrSC treated with 100 ng ml-1 rFABP4. IL-8 blocking Ab: conditioned media of PrSC treated with rFABP4 in the presence of neutralizing IL-8 antibodies. IL-6 blocking Ab: conditioned media of PrSC treated with rFABP4 in the presence of neutralizing IL-6 antibodies. Ctrl mouse IgG: conditioned media of PrSC treated with rFABP4 in the presence of isotype control mouse IgG. Ctrl goat IgG: conditioned media of PrSC treated with rFABP4 in the presence of isotype control goat IgG. (F) The relative value (%) of *in vitro* Matrigel invasion assay under each condition is shown based on the condition with PrSC cells cultured in the lower chamber. PC-3: PC-3 cells were placed in the upper chamber. PC-3 siFABP4: FABP4 siRNA1 treated PC-3 cells were placed in the upper chamber. PrSC: PrSC cells were seeded in the lower chamber. IL-8 blocking Ab, IL-6 blocking Ab, Ctrl mouse IgG, and Ctrl goat IgG as shown in (E). Bar plots represent the percentage of invading cells relative to the invasion of PC-3 with PrSC cells cultured in the lower chamber. Mean ± S.D., ^*^P < 0.05, ^**^P < 0.01.

We examined FABP4 expression and found that FABP4 was highly expressed in PCa PC-3 cells cultured for 24 hours, but not in LNCaP and DU145 cells (Supplementary Figure 1A). The FABP4 concentration in the conditioned medium (CM) of PCa cells obtained from the above experiments (measured by a human FABP4 specific ELISA kit) showed a high amount of FABP4 in PC-3 CM, but not in CM from LNCaP and DU145 cells (Supplementary Figure 1B). In addition, FABP4 expression was significantly decreased 5.1–7.5-fold by western blotting analysis (*P*=0.005), and was decreased 4.0–5.2-fold by ELISA (*P*=0.007) in PC-3 cells and PC-3 CM by treatment with 50 nM FABP4 specific siRNA-1 compared with control PC-3 cells (Figure 1A and 1B). These findings suggest that the adipokine FABP4 is highly expressed and secreted in some types of PCa such as PC-3 cells.

Next, to address the functional role of FABP4 secreted by PC-3 cells in the PCa stroma, prostate stromal cells (PrSC) were treated with recombinant FABP4 (rFABP4) or PC-3 CM, and secreted cytokines were measured by a Human cytokine array for Interleukin (IL)-8, IL-1β, IL-6, IL-10, TNFα, and IL-12p70. IL-8 and IL-6 levels were increased 7.9- and 10.6-fold (*P*=0.0003 and *P*=0.0002, respectively) in the CM of PrSC treated with 100 ng ml^-1^ rFABP4 compared with untreated PrSC CM. IL-8 and IL-6 levels were also increased 3.0- and 2.6-fold (*P*=0.004 and *P*=0.007, respectively) in the CM of PrSC treated PC-3 CM compared with untreated PrSC CM. These effects were markedly abrogated by the addition of 30 μM of a FABP4 specific inhibitor, BMS309403 (Figure 1C). In addition, cytokine production was significantly decreased in PrSC stimulated with CM from PC-3 cells treated with FABP4 siRNA-1 compared with control PC-3 CM (*P*=0.006 and *P*=0.003, respectively, Figure 1C). Although PC-3 and DU145 cells also secreted low amounts of IL-8 and IL-6, the cytokine levels were not affected by treatment with rFABP4 or FABP4 siRNA-1 (Supplementary Figure 2A and 2B). In addition, the expression of alpha smooth muscle actin (αSMA), a stromal activation marker, was upregulated by treatment with 100 ng ml^-1^ rFABP4 or PC-3 CM (2.7- and 2.1-fold, respectively), and the effects were blocked by adding 30 μM BMS309403 (1.0- and 0.9-fold, respectively) (Figure 1D). Although some of these data were not evaluated by statistical analysis, the results suggest that FABP4 secreted by some PCa cells may stimulate surrounding prostate stromal cells to produce proinflammatory cytokines in the prostate stromal tumor microenvironment.

To address the functional role of FABP4 in the activation of PrSC, we performed a MTT-based cell proliferation assay and Matrigel invasion assay. No significant differences were observed in the proliferation rate between PC-3 and DU145 cells treated with CM from PrSC cultured with or without 100 ng ml^-1^ FABP4 for 24 hours compared with untreated PC-3 and DU145 cells (Supplementary Figure 3A). The invasive capacity of PC-3 cells was significantly enhanced when they were cultured in medium containing CM from PrSC treated with 100 ng ml^-1^ of rFABP4 (PrSC-rFABP4 CM) compared with PC-3 cells cultured in CM from untreated PrSC (PrSC CM) or control medium (Ctrl) (*P*=0.0031 and *P*=0.0004, respectively). These effects were attenuated by the addition of 10 μg ml^-1^ IL-8 or 10 μg ml^-1^ IL-6 capture antibodies, but not isotype control antibodies (Figure 1E). Similarly, PC-3 cells showed increased invasive capacity when directly culturing PrSC in the lower chamber of a Matrigel invasion assay system, but this effect was significantly abrogated in PC-3 cells treated with 50 nM FABP4 siRNA-1 (Figure 1F). In addition, PrSC-rFABP4 CM and PrSC CM also significantly stimulated DU145 cell invasiveness (*P*=0.009 and *P*=0.024, respectively, Supplementary Figure 3B). These findings suggest that PrSC enhances PCa cell invasiveness by secreting IL-8 and IL-6 in response to FABP4 secreted by PCa cells.

### FABP4 directly stimulates PCa cell invasiveness by upregulating matrix metalloproteinases 2 and 9

**Figure 2:**
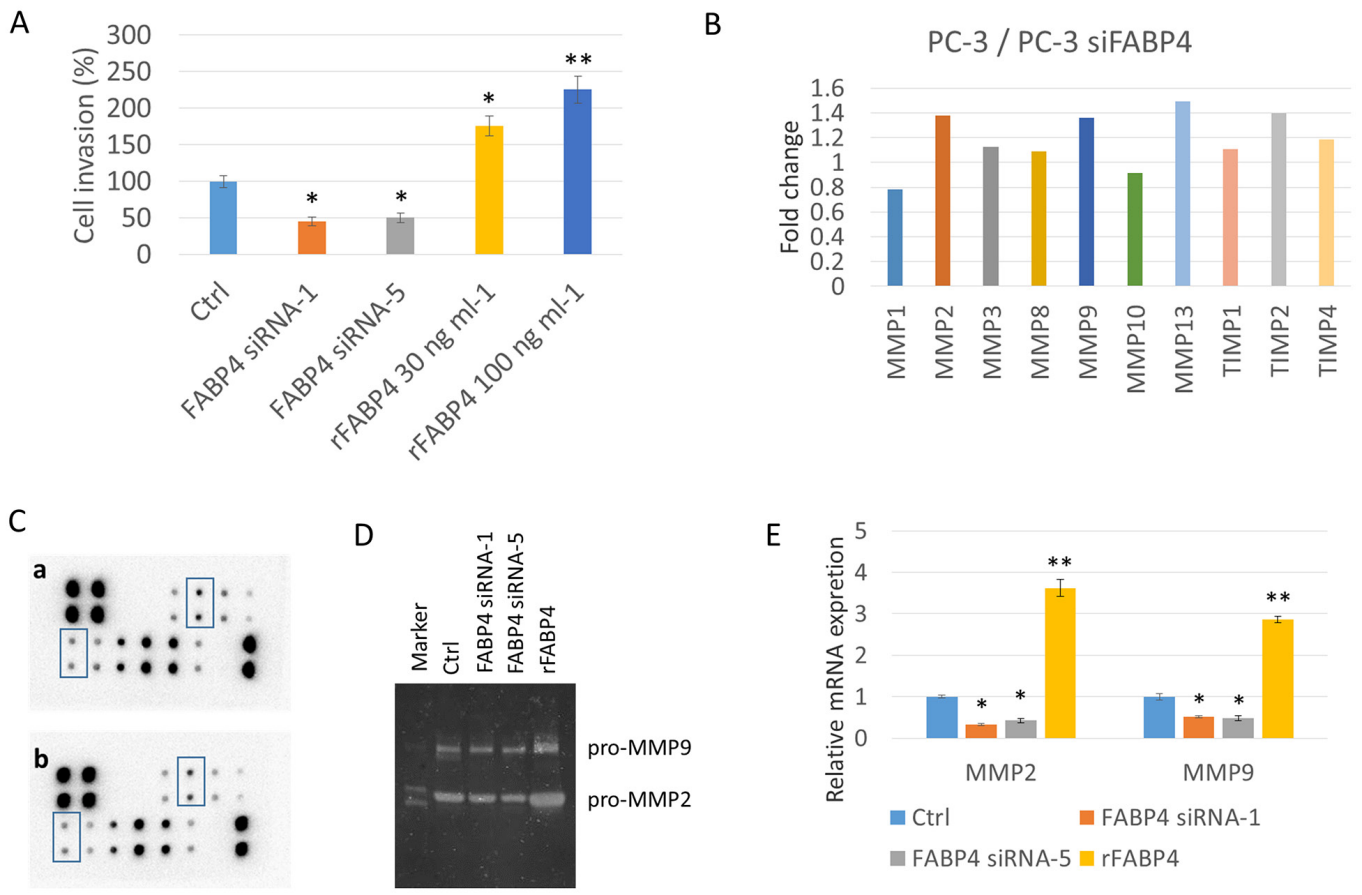
FABP4 directly stimulates PCa cell invasiveness by the upregulation of MMP2 and MMP9 **(A)** Histogram showing the relative value (%) of invading cells of each condition based on untreated PC-3 cells. PC-3 cells were treated with 50 nM FABP4 siRNAs or rFABP4 at the indicated concentration. All invading cells were counted and compared with untreated PC-3 cells (Ctrl); ^*^P < 0.05 and ^**^P < 0.01. **(B, C, D** and **E)** Altered matrix metalloproteinase (MMPs) levels by FABP4. (B and C) Overall, 1 × 10^5^ PC-3 cells were cultured in a 35-mm dish, and treated with 50 nM FABP4 siRNA-1. Each conditioned medium (CM) was then collected and analyzed for MMP levels using the RayBio Human MMP Antibody Array kit. The signal intensity in the array images was quantified and scored by Densitograph software (ATTO). Fold changes among samples were analyzed and compared using RayBio Antibody Array Analysis Tool provided by RayBiotech (B). MMP2 and MMP9 signals in the array images are indicated by rectangular boxes (C). Fold changes of signals in control PC-3 cells (upper) and FABP4 siRNA-1 treated PC-3 cells (lower) were analyzed using the RayBio Antibody Array Analysis Tool (C). (D) Zymography analysis of MMP2 and MMP9 activity. MMP2 and MMP9 enzymatic activity in condition medium from PC-3 cells treated with 50 nM FABP4 siRNAs or 100 ng ml^-1^ rFABP4. (E) Relative MMP2 and MMP9 mRNA levels. PC-3 cells were treated with 50 nM FABP4 siRNAs and 100 ng ml^-1^ rFABP4 for 24 hours. *MMP2, MMP9* and *beta-actin* mRNA levels were assessed by quantitative RT–PCR, and compared with untreated cells. Mean ± S.D., ^*^P < 0.05 and ^**^P < 0.01.

To investigate the direct role of FABP4 in PCa cell proliferation and invasion, we compared the proliferation rate and invasive capacity of PC-3 cells treated with FABP4 siRNAs (50 nM) or rFABP4 (30 and 100 ng ml^-1^) with that of untreated PC-3 cells. There was no significant difference in the proliferation rate of PC-3 cells after 24 hours administration of FABP4 siRNAs or rFABP4 compared with untreated PC-3 cells (Supplementary Figure 4). In contrast, siRNA-mediated knockdown of FABP4 significantly reduced the invasive capacity of PC-3 cells (*P*=0.031 and *P*=0.015, respectively), and rFABP4 significantly increased the invasive capacity of PC-3 cells in a dose-dependent manner (*P*=0.028 and *P*=0.007, respectively) (Figure 2A). Interestingly, although FABP4 was not expressed in DU145 cells, rFABP4 also stimulated DU145 cell invasiveness (Supplementary Figure 5A).

Next, we tried to clarify the underlying mechanism of FABP4 mediated enhanced invasiveness of PC-3, and screened matrix metalloproteinases (MMP) expression in CM from PC-3 cells treated with FABP4 siRNA-1 and control CM from PC-3 cells using a Human MMP antibody array. Compared with the CM of PC-3 cells treated with FABP4 siRNA-1, the control PC-3 CM had increased levels of MMP2 (1.38-fold), MMP9 (1.36-fold), MMP13 (1.49-fold) and tissue inhibitor of metalloproteinase 2 (TIMP2; 1.40-fold) (Figure 2B and 2C: rectangular boxes in the array image). In addition, gelatin zymography demonstrated the protein expression of pro-MMP2 and 9 was lower in the CM of PC-3 cells treated with FABP4 siRNAs compared with CM from control PC-3 cells (FABP4 siRNA1: 0.45- and 0.40-fold; FABP4 siRNA2: 0.52- and 0.54-fold, respectively), and the expression of pro-MMP2 and 9 was increased in the CM from PC-3 cells treated with 100 ng ml^-1^ rFABP4 (2.2- and 2.7-fold, respectively) (Figure 2D). Although these data were not evaluated by statistical analysis, the results strongly suggest that the MMPs protein expression and secretion are affected by FABP4. Furthermore, quantitative RT-PCR demonstrated the mRNA expression levels of *MMP2* and *9* were significantly downregulated in PC-3 cells by treatment with FABP4 siRNAs compared with control PC-3 cells (*P*=0.017 and *P*=0.025; *P*=0.043 and *P*=0.029, respectively), and rFABP4 significantly stimulated *MMP2* and *9* mRNA expression (*P*=0.006 and *P*=0.008, respectively) (Figure 2E). Interestingly, FABP4 also significantly enhanced the mRNA expression of *MMP2* and *9* in DU145 cells (Supplementary Figure 5B). These findings strongly suggest that FABP4 directly stimulates PCa cell invasiveness by upregulating MMPs.

### Akt and Erk signaling pathways are required for FABP4-induced PrSC activation and upregulation of PCa cell invasiveness

**Figure 3:**
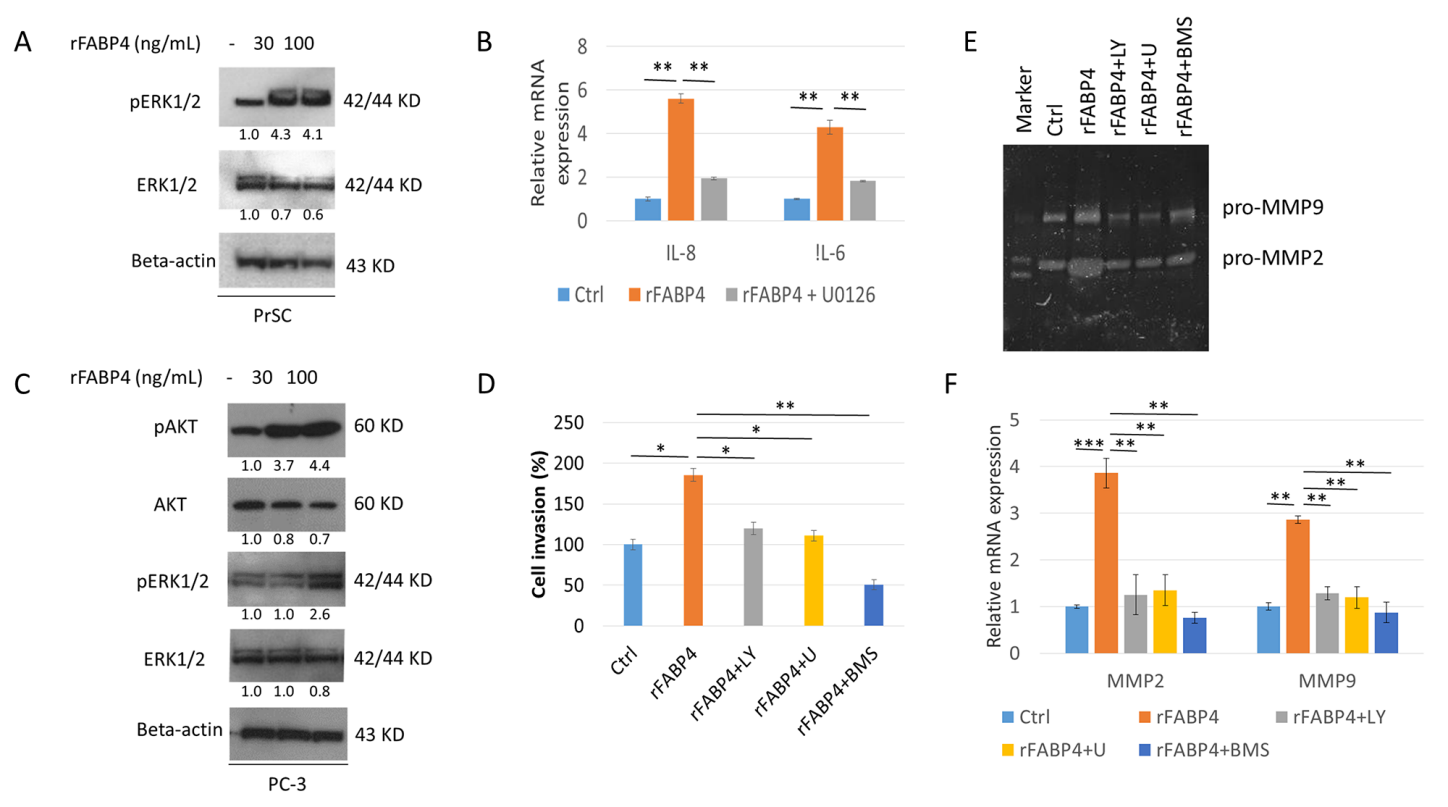
AKT and ERK signaling pathways are required for FABP4 induced PrSC activation and upregulation of PCa cell invasiveness **(A** and **B)** Requirement of ERK activation for enhanced IL-8 and IL-6 in PrSC by FABP4. PrSC cells were treated with rFABP4 at 30 and 100 ng ml^-1^ for 3 hours. Cells cultured previously in the presence or absence of 1 μM U0126 for 1 hour before treatment with rFABP4 (A), *IL-8* and *IL-6* mRNA levels were measured by quantitative RT-PCR, and compared with untreated cells (B). ^**^P < 0.01. **(C)** AKT and ERK activation of PC-3 by FABP4. PC-3 cells were treated with 0–100 ng ml^-1^ rFABP4 for 3 hours. Then, an equal amount of protein (10 μg) from the cells was subjected to anti-AKT, anti-pAKT, anti-ERK, anti-pERK, and anti-beta-actin antibodies. **(D)** Requirement of AKT and ERK activation for enhanced invasion of PC-3 cells by FABP4. The relative value (%) of *in vitro* Matrigel invaded PC-3 cells is shown based on untreated PC-3 cells. rFABP4: PC-3 cells treated with 100 ng ml^-1^ rFABP4 for 24 hours. The cells were treated with 10 μM LY294002 (rFABP4+LY) or 1 μM U0126 (rFABP4+U) for 1 hour before treatment with rFABP4. rFABP4+BMS: PC-3 cells treated with 100 ng ml^-1^ rFABP4 for 24 hours with 30 μM BMS309403. Then, all invading cells were counted and compared with untreated cells; ^*^P < 0.05 and ^**^P < 0.01. **(E** and **F)** Requirement of AKT and ERK activation for MMP activation of PC-3 cells by FABP4. PC-3 cells were treated with 100 ng ml^-1^ rFABP4 alone (rFABP4) and 10 μM LY294002 (rFABP4+LY), 1 μM U0126 (rFABP4+U), or 30 μM BMS309403 (rFABP4+BMS). (E) MMP2 and MMP9 enzymatic activity in culture medium was determined by Zymography analysis. (F) Relative values of *MMP2* and *MMP9* mRNA levels were measured by quantitative RT-PCR, and the levels were compared with untreated cells. ^**^P < 0.01 and ^***^P < 0.001.

To delineate the expression and activation of protein kinase pathways in FABP4-induced PrSC activation and PCa cell invasiveness, we investigated the phosphatidylinositol 3-kinase (PI3K) and mitogen-activated protein kinase (MAPK) signaling pathways that were reported to be involved in prostate carcinogenesis [37, 38]. Phospho-ERK1/2 (pERK1/2), and especially pERK1 expression, was increased in PrSC treated with rFABP4 at 30 and 100 ng ml^-1^ compared with baseline values (4.3- and 4.1-fold, not statistically evaluated) (Figure 3A). In addition, *IL-8* and *IL-6* mRNA expressions were markedly increased in PrSC by treatment with 100 ng ml^-1^ rFABP4 and attenuated by pretreatment with 1 μM U0126, an ERK kinase inhibitor (Figure 3B). However, phospho-AKT (pAKT) protein expression in PrSC was not affected by rFABP4 treatment (data not shown). The expressions of pAKT and pERK were upregulated in PC-3 cells treated with 100 ng ml^-1^ rFABP4 (4.4- and 2.6-fold, not statistically evaluated) (Figure 3C). In addition, the invasive capacity of PC-3 cells was significantly increased by treatment with 100 ng ml^-1^ rFABP4 (*P*=0.035), and the effect was abrogated by pretreatment with 10 μM of a PI3K inhibitor, LY294002 (*P*=0.025), 1 μM of an ERK inhibitor, U0126 (*P*=0.037) or 30 μM of a FABP4 inhibitor, BMS309403 (*P*=0.008) (Figure 3D). Moreover, the increased expression levels of MMP2 and 9 proteins and mRNAs in PC-3 cells treated with 100 ng ml^-1^ rFABP4 were downregulated by pretreatment with 10 μM LY294002 (*P*=0.007 and *P*=0.003, respectively), 1 μM U0126 (*P*=0.003 and *P*=0.008, respectively), or 30 μM BMS309403 (*P*=0.002 and *P*=0.007, respectively) (Figure3E and 3F). These findings suggest that Akt and Erk signaling pathways have a critical role in the FABP4-mediated activation of prostate stromal cells and PCa cell invasiveness.

### PrSC or HFD conditions increase PCa invasiveness *in vivo*

**Figure 4:**
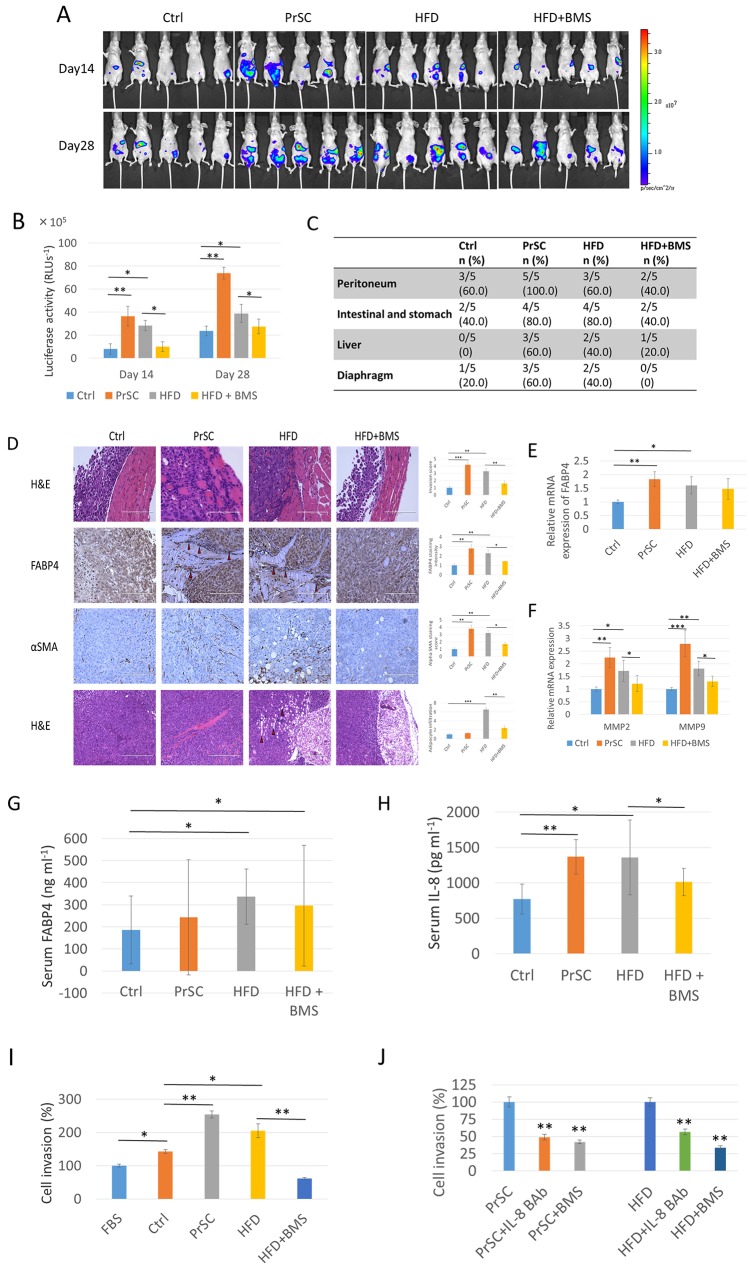
PrSC or HFD conditions stimulate PCa metastasis and invasiveness *in vivo* For *in vivo* metastatic tumor studies, PC-3M-luc-C6 cells were intraperitoneallyinjected into mice randomly assigned to four groups (5 mice per group): control (Ctrl), PrSC, HFD, and HFD+BMS. For the PrSC group, PrSC cells were injected with the tumor cells. Mice in the HFD and HFD+BMS groups were fed HFD, and mice in the Ctrl and PrSC groups were fed a control diet. Mice in the HFD+BMS group were administrated BMS309403 by dissolution in drinking water. **(A** and **B)** Increased luciferase activity by PrSC or HFD. At 14 and 28 days inoculation, bioluminescence was used to detect intraperitoneal tumor growth and metastases by the intraperitoneal injection of luciferin. **(C)** Increased tumor metastasis in the PrSC and HFD groups. At 29 days after the injection of cells, mice were sacrificed, and the proportion of metastatic tumor burden in peritoneal organs, peritoneum, intestine, stomach, liver, and diaphragm were evaluated. **(D, E** and **F)** Increased invasive capacity in the PrSC and HFD groups by the increased adipocyte infiltration, stromal fibroblast activation, and upregulation of FABP4 and MMPs. (D) Slides of mouse tumor tissues were subjected to hematoxylin and eosin staining (H & E), and immunohistochemistry to detect the expression of FABP4 and αSMA. (E and F) mRNA expressions of *FABP4* and *MMP2* and *9* in tumors from each group were analyzed by quantitative RT-PCR. The mRNA expression levels of *FABP4* and *MMP2* and *9* was normalized to the levels of *beta-actin*, and the relative values were compared with the level of the Ctrl group. ^*^P < 0.05, ^**^P < 0.01, and ^***^P < 0.001. **(G)** HFD stimulated FABP4 expression. The serum FABP4 concentration was measured by a mouse FABP4 ELISA kit. The serum FABP4 level was significantly higher in the HFD and HFD+BMS groups compared with the Ctrl group. ^*^P < 0.05. **(H)** Increased serum IL-8 levels in the PrSC and HFD groups. The serum cytokine concentrations were measured by a Cytometric bead array kit. The serum level of IL-8 was significantly higher in the PrSC and HFD groups compared with the Ctrl group, and in the HFD group compared with the HFD+BMS group. ^*^P < 0.05, ^**^P < 0.01. **(I** and **J)** Stimulation of PC-3 cell invasiveness by mouse serum containing higher concentrations of FABP4 and IL-8. *In vitro* cell invasion is shown (I), and in the presence of 30 μM BMS309403 or 10 μg ml^-1^ IL-8 blocking antibody (J). All invading cells were counted and compared with control cells. Mean ± S.D., ^*^P < 0.05, ^**^P < 0.01.

Because circulating levels of FABP4 are associated with obesity and metabolic diseases, we investigated the relationship between HFD, PrSC activation and PCa progression in an *in vivo* model. We inoculated 2 × 10^6^ PC-3M-lu c-C6 cells with stable luciferase expression intraperitoneally to 8-week old BALB/c-nu/nu mice. FABP4 was highly expressed in PC-3M-luc-C6 and PC-3 cells (Supplementary Figure 6). Mice were randomly assigned to four groups (5 mice per group): control (Ctrl), PrSC, HFD, and HFD with BMS309403 administration (HFD+BMS) groups. For the PrSC group, 2 × 10^5^ PrSC were intraperitoneally injected with PC-3M-luc-C6 cells. Mice in the HFD and HFD+BMS groups were fed with HFD, and mice in the Ctrl and PrSC groups were fed with control food (CE-2 diet). The FABP4 inhibitor BMS309403 was administered to mice in the HFD+BMS group by dissolution in drinking water at 40 μg ml^-1^ throughout the dietary experiments. There were no statistical differences in mouse weight in each group at the end of the experiments (data not shown). The extent of the tumor burden was detected using the Xenogen IVIS™ imaging system at 14 and 28 days after inoculation of the cells into mouse peritoneum. The tumor burden was higher in the PrSC and HFD groups compared with the Ctrl group, and in the HFD group compared with the HFD+BMS group (Figure 4A). The luciferase activity (60-s exposure) of each mouse was measured at 14 and 28 days after inoculation of the cells, and the level of luciferase activity was significantly higher in the PrSC and HFD groups compared with the Ctrl group (*P*=0.004 and *P*=0.019; and *P*=0.006 and *P*=0.033, respectively), and was markedly higher in the HFD group compared with the HFD+BMS group (*P*=0.014 and *P*=0.038, respectively) (Figure 4B). PC-3M-luc-C6 cells efficiently formed tumors in the peritoneal organs including the peritoneum, intestine, stomach, liver, and diaphragm. The rate of tumor formation in the peritoneal organs was increased in the PrSC group compared with the Ctrl group and in the HFD group compared with the HFD+BMS group, and the total metastasis rates were calculated (*P*=0.003 in the PrSC group compared with the Ctrl group, and *P*=0.028 and *P*=0.034 in the HFD group compared with the Ctrl and HFD+BMS groups, respectively Figure 4C). Hematoxylin and eosin staining showed a higher tumor cell transmigration into the peritoneum and adipocyte infiltration in the tumor microenvironment in the PrSC and HFD groups compared with the Ctrl and HFD+BMS groups (Figure 4D). As indicated by the red arrow in Figure 4D, a higher FABP4 staining intensity was observed in tumor cells at the surface between invasive cancer cells and the peritoneum tissue of mice in the PrSC and HFD groups. The mRNA expression level of *FABP4* was also significantly higher in the peritoneal tumors of the PrSC and HFD group compared with the Ctrl group (*P*=0.002 and *P*=0.016, respectively) (Figure 4E). The expression of αSMA, a specific marker of reactive fibroblasts, was higher in tumors of the PrSC and HFD groups (Figure 4D). The expressions of vimentin and cytokeratin (AE1/AE3), fibroblast and epithelial tumor markers, respectively, were high in the peritoneal tumors of each group (Supplementary Figure 7A and 7B). Interestingly, the HFD consumption significantly stimulated adipocyte infiltration in the tumor tissue, and the effect was abrogated by the administration of BMS309403, a FABP4 specific inhibitor (Figure 4D). The mRNA expressions of *MMP2* and *9* were significantly higher in peritoneal tumors of the PrSC group compared with the Ctrl group (*P*=0.0061 and *P*=0.0003, respectively), and were also significantly higher in the HFD group compared with the Ctrl and HFD+BMS groups (*P*=0.025 and *P*=0.042; and *P*=0.003 and *P*=0.042, respectively) (Figure 4F). The mean ± standard deviation serum levels of FABP4 were 191.9 ± 99.8, 250.1 ± 217.4, 354.2 ± 123.3, and 296.3 ± 159.5 ng ml^-1^, in the Ctrl, PrSC, HFD, and HFD+BMS groups, respectively. The serum FABP4 level was significantly higher in the HFD group compared with the Ctrl group (*P* < 0.05), and in the HFD+BMS group compared with the Ctrl group (*P* < 0.05, Figure 4G). Moreover, the mean IL-8 levels measured by Cytokine array were 773.2 ± 210.7, 1370.9 ± 243.6, 1358.9 ± 528.5, and 1015.3 ± 192.8 pg ml^-1^ in the Ctrl, PrSC, HFD, and HFD+BMS groups, respectively. The mean IL-8 level was significantly higher in the PrSC group compared with the Ctrl group (*P*=0.007), and in the HFD group compared with the Ctrl and HFD+BMS groups (*P*=0.022 and *P*=0.036, respectively) (Figure 4H). The serum level of IL-6 was too low in each group to make an objective comparison.

To determine whether increased serum levels of FABP4 and IL-8 modified tumor cell proliferation and invasive capacity, we performed *ex vivo* proliferation and invasion assays using serum harvested from mice. The proliferation rate of PC-3 cells was not statistically different between each group in the presence of 5% mouse serum obtained from each group. The invasive capacity was significantly increased in PC-3 cells cultured in 5% mouse serum from PrSC compared with the Ctrl group (*P*<0.01), and in the serum from the HFD group compared with the Ctrl and HFD+BMS groups, respectively (*P*<0.05 and *P*<0.01) (Figure 4I). Furthermore, the increased effects were markedly abrogated by the presence of 10 μg ml^-1^ IL-8 blocking antibody or 30 μM BMS309403 (*P*<0.01 and *P*<0.01 in the PrSC group, and *P*<0.01 and *P*<0.01 in the HFD group, respectively; Figure 4J). These results strongly suggest that PrSC or HFD consumption stimulates PCa invasiveness by the upregulation and secretion of FABP4 and IL-8.

### Overexpression and secretion of FABP4 is associated with human PCa progression

**Figure 5:**
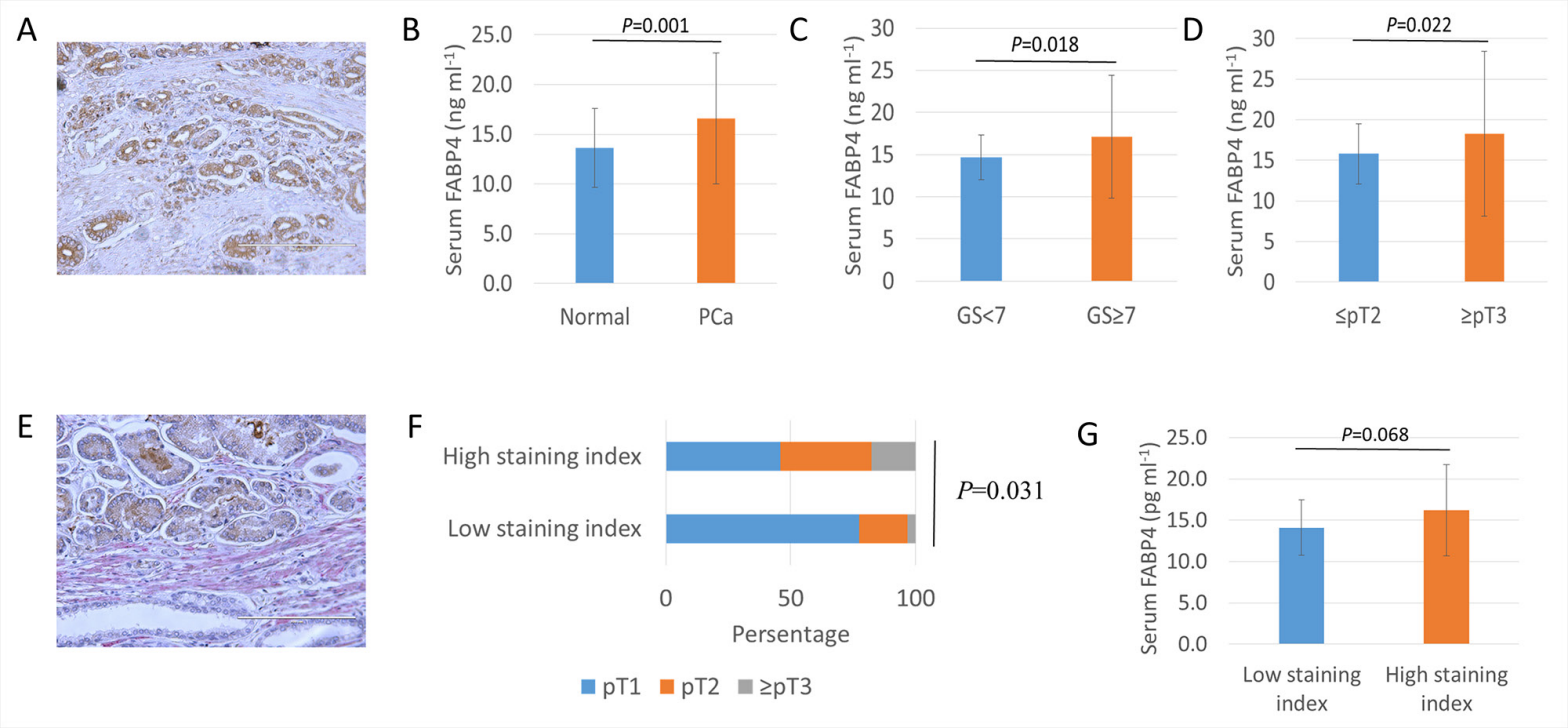
Overexpression and secretion of FABP4 is associated with PCa progression **(A)** Immunohistology staining of tissue samples from PCa radical prostatectomy using an anti-human FABP4-specific antibody. **(B, C,** and **D)** Serum FABP4 levels from 40 controls and 104 patients with PCa were measured using a human FABP4 ELISA Kit. (B) The serum FABP4 level was significantly higher in PCa patients than in the controls (16.6 ± 6.6 and 13.6 ± 4.0 ng ml^-1^, respectively, *P* = 0.001). (C) The serum FABP4 level was significantly higher in patients with a GS of ≥ 7 (*n*=79) compared with a GS of ≤ 6 (*n*=25) (17.1 ± 7.2 and 14.6 ± 2.6 ng ml^-1^, respectively, *P* = 0.018). (D) The serum FABP4 level in surgical specimens was significantly higher in patients with pathological T (pT) stages (≥ pT3, *n*=34) of PCa than in those with more localized disease (≤ pT2, *n*=70) (18.2 ± 10.1 and 15.8 ± 3.7 ng ml^-1^, respectively, *P* = 0.022). **(E)** The percentage of αSMA-positive staining stromal cells in the surrounding of cancer cells was calculated, and the staining level was determined by the staining percentage score characterized as low-staining (≤10%) and high-staining (>10%). **(F)** The relationship between αSMA expression level and pathological T stage (pT) was evaluated. Patients with low αSMA staining were comprised 77.4% of pT1, 19.4% of pT2, and 3.2% of pT3 or 4. Patients with high αSMA staining were comprised 45.9% of pT1, 36.5% of pT2, and 17.6% of pT3 or 4 (*P* = 0.031). **(G)** The serum FABP4 level was higher in patients with high αSMA staining compared with low αSMA staining (FABP4, 14.8 ± 0.3 and 16.2 ± 0.5 ng/ml, *P* = 0.068; MIC-1, 891.4 ± 218.4 and 1075.9 ± 354.8 pg/ml, *P* = 0.056, respectively).

**Figure 6:**
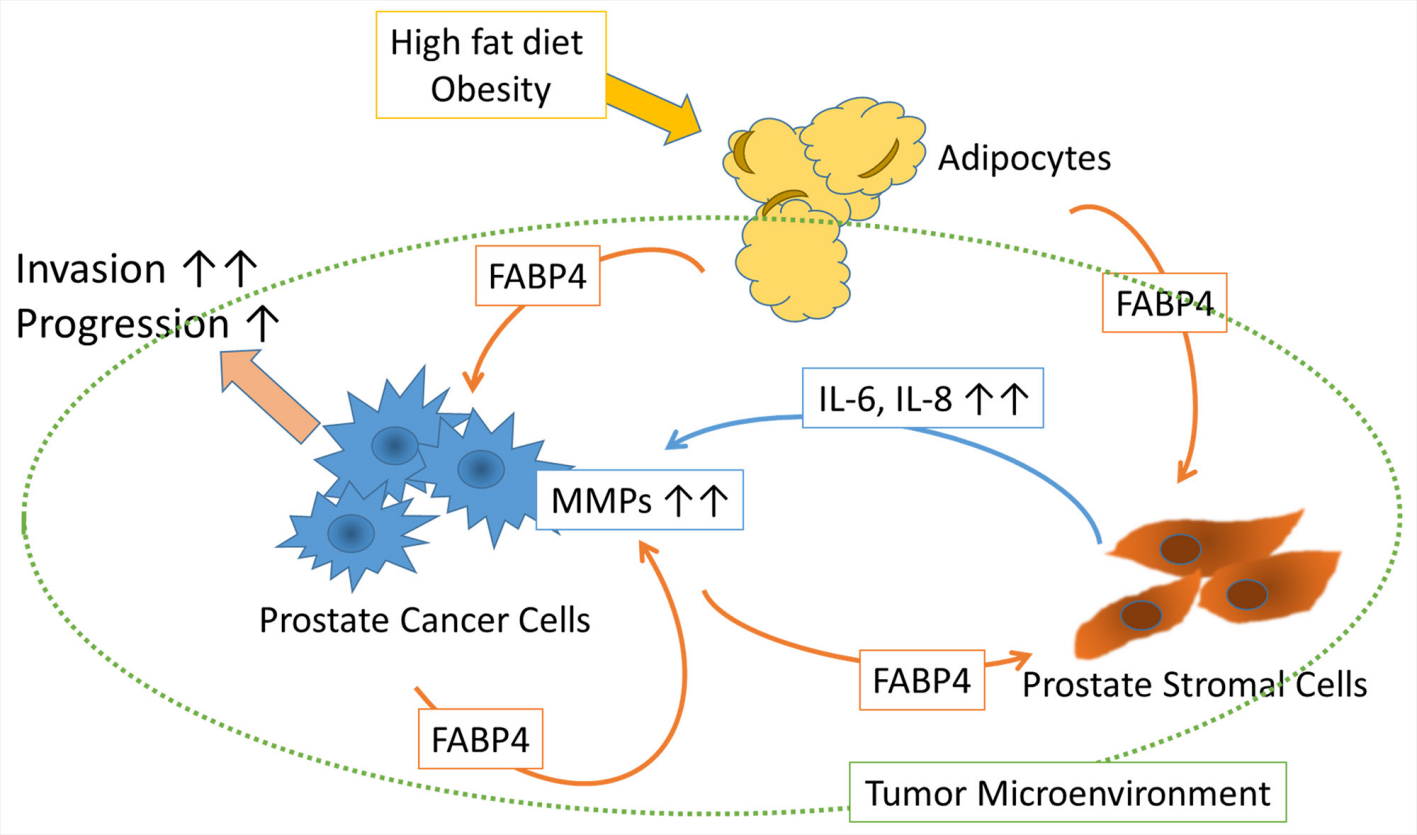
Schematic of the putative effect of FABP4 on the tumor microenvironment FABP4 secreted from PCa cells directly stimulates PCa cell invasiveness via the upregulation of MMPs. FABP4 activates PrSCs resulting in the augmentation of PCa metastasis and invasiveness via the secretion of IL-8 and IL-6. HFD influences PCa metastasis and invasiveness by upregulating FABP4 and IL-8, suggesting circulating FABP4 might have an important role in PCa progression.

To determine the role of FABP4 on the generation of reactive PCa stroma and the related clinical PCa progression, we performed FABP4 immunohistochemistry in specimens from 104 PCa patients treated by radical prostatectomy. FABP4 was predominantly expressed in cancer epithelial cells, but there was no significant correlation between FABP4 staining level and clinical parameters (Figure 5A). We measured serum FABP4 levels in 40 healthy controls and 104 PCa patients, and found the mean serum FABP4 level was significantly higher in PCa patients compared with the controls (16.6 ± 6.6 and 13.6 ± 4.0 ng ml^-1^, respectively, *P*=0.001; Figure 5B). The relationship between serum FABP4 and Gleason score (GS) was significantly higher (*P*=0.018; Figure 5C) in PCa patients (17.1 ± 7.2 ng ml^-1^; GS ≥ 7; *n*=79) compared with controls (14.6 ± 2.6 ng ml^-1^; GS ≤ 6; *n*=25). In addition, the serum FABP4 level was significantly higher (*P*=0.022; Figure 5D) in patients with advanced pathological T (pT) PCa stage (≥ pT3; 18.2 ± 10.1 ng ml^-1^; *n*=34) compared with those with a more localized disease (≤ pT2; 15.8 ± 3.7 ng ml^-1^; *n*=70). However, there was no significant association between FABP4 levels and the body mass index (BMI) or age in PCa patients. For example, the mean FABP4 level was 16.5 ± 7.3 ng ml^-1^ in PCa patients with a BMI < 25 (*n*=76) and 16.8 ± 4.0 ng ml^-1^ in patients with a BMI ≥ 25 (*n*=28) (*P*=0.471, Supplementary Figure 8). In addition, αSMA was predominantly expressed in the PCa stroma, and the staining level of αSMA in PCa specimens was calculated according to the proportion of αSMA-positive cells (Figure 5E). Patients with low levels of αSMA staining comprised 77.4% of pT1, 19.4% of pT2, and 3.2% of pT3 or 4, and patients with high levels of αSMA staining comprised 45.9% of pT1, 36.5% of pT2, and 17.6% of pT3 or 4. Thus, high levels of αSMA staining were markedly superior in numbers and percentage in patients with advanced pT stages (≥pT2) of PCa than that of low αSMA staining (*P*=0.031, Figure 5F). However, there was no statistical difference in the staining level of αSMA with GS score (*P*=0.323) or BMI (*P*=559). In addition, the serum level of FABP4 was higher in patients with high levels of αSMA staining compared with patients with low levels of αSMA staining (*P*=0.068, Figure 5F). These findings suggest that the high expression of FABP4 in tumors and increased serum FABP4 levels may be associated with PCa progression through the generation of reactive PCa stroma in PCa patients rather than the extent of obesity (Figure 6).

## DISCUSSION

In the present study, we investigated the expression and functional role of FABP4 in PCa, and found that FABP4 directly and/or indirectly stimulated PCa cell invasiveness, both *in vitro* and *in vivo*. FABP4 is one of the most abundant proteins in adipocytes, and its expression and secretion were affected by multiple networks, common related factors including insulin and/or insulin like growth factor-1 (IGF-1), fatty acids, and nucleus receptor PPAR γ [17, 18, 22, 24], as well as HFD and dietary components [17, 19, 20]. In addition, FABP4 present in various types of cancer cells was involved in cancer progression [32-34]. The present study demonstrated that FABP4 was highly expressed in cultured PCa cells and then secreted into CM. In human PCa and serum, FABP4 was overexpressed and elevated. In addition, FABP4 promoted PCa invasiveness not only by the stimulation of PrSC to produce IL-8 and IL-6, but also by directly stimulating the upregulation of MMPs through activation of the PI3K/AKT and ERK signaling pathways. Moreover, the HFD induced upregulation of FABP4 also promoted PCa growth and invasiveness *in vivo*, in an animal model. This effect was downregulated by the addition of a FABP4 inhibitor. These findings strongly suggest that FABP4 may have a critical role in PCa progression, especially under conditions of obesity or HFD (Figure 6).

PrSC are a major component of the prostate stroma. They have an important role in PCa progression via crosstalk with adjacent cancer cells to secrete proinflammatory cytokines and chemokines, such as IL-8, IL-6 and CXCLs in response to secreted factors from prostate epithelia and cancer cells. These increased cytokines may be an important therapeutic target for PCa [12, 14]. In addition, PTEN loss upregulated FABP4 expression in keratinocytes [29], and its loss sustained the growth and survival of PTEN-deficient prostate epithelium by inducing a selective upregulation of IL-8 signaling [39]. Adipocytes are an important component of the tumor microenvironment, and promote ovarian cancer metastasis by the upregulation of FABP4 and IL-8 [35]. A previous report showed that obesity-induced periprostatic adipocytes facilitated the extraprostatic extension of PCa cells by enhanced CCR3/CCL7 signaling [16]. In the present study, PrSC were activated by FABP4 secreted from PCa cells and PrSC stimulated IL-8 and IL-6 secretion to promote PCa cell invasiveness. In addition, HFD also significantly increased the serum level of FABP4 and IL-8, and promoted PCa metastasis and invasiveness in an animal xenograft model, although the origin of the high serum FABP4 and IL-8 levels is not clear. Furthermore, secreted FABP4 directly enhanced the invasive potential of PCa cells by upregulating MMP2 and MMP9 expression possibly in an autocrine and paracrine manner. Higher circulating FABP4 levels were associated with obesity and other metabolic syndromes [40]. These findings indicate that FABP4 is a secretory and regulatory factor that mediates the synergistic activation of PCa stroma and PCa cells critically involved in PCa progression, especially under conditions of HFD or obesity. In this study, the mean serum FABP4 level of PCa patients was not associated with BMI, but was associated with advanced PCa disease status. This implies that serum FABP4 is influenced by disease status rather than obesity. However, the origin of the high serum FABP4 levels (i.e., cancer cell origin or fat tissue origin) remains to be determined. In addition, it is unclear whether the findings in this study were related to the racial background (all subjects in this study were Japanese PCa patients).

PCa metastasis and invasiveness was stimulated by enhanced cytokine signaling through activated PI3K/AKT and ERK pathways [41, 42]. MMPs are essential in facilitating invasion and metastasis of PCa [43, 44], and previous studies, including our own, have shown that MMP expression was enhanced by various cytokines [45, 46]. In the present study, we demonstrated that FABP4 directly stimulated PCa cell invasiveness by upregulating MMPs through the activation of PI3K/AKT and ERK signaling pathways *in vitro*. Interestingly, although FABP4 was not expressed in DU145 PCa cells, extracellular FABP4 also stimulated cell invasiveness presumably by MMP upregulation (Supplementary Figure 5). FABP4 was expressed abundantly in adipocytes and was present in peripheral blood suggesting it induces the activation of MMPs, an important mechanism in PCa with FABP4 positive or negative expression. Interestingly, a recent study has shown that FABP4 has an important role in the metastatic progression of PCa in bone [20]. It would be interesting to determine whether the accelerated formation of bone metastatic lesions was mediated by enhanced MMPs.

The molecular mechanisms underlying the expression and secretion of FABP4 in PCa cells is unclear. In the present study, FABP4 was strongly expressed and secreted by PC-3 cells, but not by LNCaP and DU145 cells. Interestingly, the overexpression of androgen receptor (AR) in PC-3 cells decreased the expression of PPAR gamma, followed by a decrease in FABP4 [25]. In addition, the expression of PTEN, a major PCa tumor suppressor that interacts with FABP4, was inversely correlated with FABP4 expression [28, 29]. These findings suggest FABP4 expression and secretion are associated with the AR and PTEN pathways and correlate with PCa progression.

BMS309403 is a biphenyl azole inhibitor specifically designed to target FABP4 and it protected against severe atherosclerosis and type 2 diabetes [30]. A recent studyhas shown that BMS309403 activates AMP-activated protein kinase (AMPK) [47], which has an important role in HFD induced LNCaP xenograft progression by altering fatty acid synthase [11, 48]. In the present study, BMS309403 inhibited cytokine secretion in PrSC stimulated with FABP4. Furthermore, adipocyte infiltration into the tumor microenvironment was inhibited in mice orally administered BMS309403, which also protected against PCa xenograft growth and local or metastatic PCa expansion stimulated by HFD. These findings strongly suggest that BMS309403 may be a useful drug for chemoprevention and therapy in PCa. Furthermore, it would be interesting to investigate the interactions of FABP4 and PrSC *in vivo* by preparing PrSC with BMS309403 group with or without HFD.

In conclusion, we found that FABP4 was highly expressed and secreted by PCa cells, and that FABP4 directly stimulated PCa cell invasiveness by the upregulation of MMPs through the activation of Akt and Erk signaling pathways (schematic shown in Figure 6). In addition, FABP4 activated PrSCs, resulting in the secretion of IL-8 and IL-6 and the subsequent augmentation of PCa metastasis and invasiveness. Furthermore, HFD influenced PCa metastasis and invasiveness by the upregulation of FABP4 and IL-8 in a mouse PC-3 xenograft model. Clinically, the overexpression of FABP4 was significantly involved with an aggressive type of PCa. Although the contribution of circulating FABP4 from adipocytes to PCa cells and the tumor microenvironment is not clear from our study, our animal study with HFD suggests they are involved (Figure 6). In conclusion, FABP4 and its related prostate microenvironment may be important targets for chemoprevention and therapy in aggressive PCa.

## MATERIALS AND METHODS

### Cell culture and reagents

Human prostate cancer (PCa) LNCaP, PC-3, and DU145 cells were purchased from the American Type Culture Collection (Manassas, VA, USA). Luciferase-expressing PC-3M-luc-C6 cell line derived from PC-3M metastatic prostate cancer cells was purchased from PerkinElmer (Waltham, MA, USA). The cells were maintained in RPMI 1640 medium or DMEM (Invitrogen, Carlsbad, CA, USA) containing 10% fetal bovine serum (FBS) and 1% penicillin–streptomycin. Normal prostate stromal cells (PrSC) and optimized culture media (Clonetics™ SCGM™ Stromal Cell Growth Medium) were purchased from Lonza (Walkersville, MD, USA). The PrSC cells derived from a 40-year-old healthy male donor were maintained as frozen stocks in liquid nitrogen and used within three to seven passages after thawing. PI3K inhibitor (LY294002) and MAPK inhibitor (U0126) were purchased from Cell Signaling Technology (Boston, MA). FABP4 inhibitor, [2′-(5-Ethyl-3,4-diphenyl-pyrazol-1-yl)-biphenyl-3-yloxy] (BMS309403) was purchased from Sigma (St Louis, MO, USA), and recombinant FABP4 was purchased from ProSpec-Tany TechnoGene Ltd. (Ness-Ziona, Israel).

### Animal study

The institutional review board of the Akita University School of Medicine approved all animal experiments. Male 8-week-old athymic BALB/c-nu/nu mice were obtained from Japan SLC (Shizuoka, Japan), and two to three animals per cage were housed in a pathogen-free environment. Animals were fed with an autoclaved CE-2 diet (Japan SLC, Japan). For *in vivo* metastatic tumor studies, 2 × 10^6^ of PC-3M-luc-C6 cells were resuspended in 0.1 ml of ice-cold PBS (Invitrogen), and inoculated by intraperitoneal injection into mice using a 27-gauge needle. The mice were randomly assigned to four groups (n = 5 per group): control (Ctrl), PrSC, high-fat diet (HFD), and HFD with BMS30093 (HFD+BMS) groups. For the PrSC group, 2 × 10^5^ of PrSC were intraperitoneally injected with PC-3M-luc-C6 cells. Mice in the HFD and HFD+BMS groups were fed with HFD (consisting of 59.9% calories from fats, 21.4% from carbohydrates, and 18.6% from proteins; Purina Mills Test Diets, Richmond, IN, USA), and mice in the Ctrl and the PrSC groups were fed with control food (CE-2 diet). The FABP4 inhibitor BMS309403 was administered to mice in the HFD+BMS group by dissolution in drinking water at 40 μg ml^-1^. The body weight and amount of food consumed were measured weekly throughout the experiments. Bioluminescence was used to follow PC-3M-luc-C6 cell metastasis after the intraperitoneal injection of luciferin (200 μl at 15 mg ml^-1^ in PBS), and the reflective luciferase activity (60-s exposure) was measured by a Xenogen IVIS™ imaging system at 14 and 28 days after inoculation of the cells. Mice were sacrificed 29 days after inoculation of the tumor cells, and the expanded tumor burdens in peritoneal organs such as the peritoneum, intestine, stomach, liver, and diaphragm were evaluated. Then, the tumors were excised, and subjected to histopathology and quantitative RT-PCR analysis. Blood was collected from the orbital sinus. Serum was separated, filtered, and stored at −80 °C until use.

### siRNA constructs

FABP4 siRNA-1 (SI00026033), siRNA-5 (SI03107237) and luciferase siRNA (SI03650353) were purchased from Qiagen (Valencia, CA, USA). Luciferase siRNA was used as a control. Transfection of siRNAs was performed using Lipofectamine^®^ 2000 (Invitrogen). Cells were cultured in a 35-mm dish and treated with siRNAs (50 nM) in reduced-serum DMEM. The knockdown of FABP4 was verified by western blotting and quantitative RT-PCR.

### Quantitative RT-PCR

Total RNA was extracted from the cultured cells and tumors using TRIzol^®^ reagent (Invitrogen). The following RT-PCR primers were used: *FABP4*, forward 5′-CCT GAA AAC TGC AGC TTC CTT C-3′, reverse 5′-GGC AAA GCC CAC TCC TAC TTC-3′; interleukin-8 (*IL-8*), forward 5′-ATG ACT TCC AAG CTG GCC GTG G-3′, reverse 5′-CAT AAT TTC TGT TTG GCG CAG TGT GG-3′; *IL-6,* forward 5′-GCT TTA AGG AGT TCC TGC-3′, reverse 5′-GGT AAG CCT ACA CTT TCC A-3′; *MMP-2,* forward 5′-GTG CTG AAG GAC ACA CTA AAG-3′, reverse 5′-ACA ACT TTG AGA AGG ATG GCA A-3′; *MMP-9,* forward 5′-ATT TCT GCC AGG ACC GCT TCT ACT-3′, reverse 5′-CAG TTT GTA TCC GGC AAA CTG GCT-3′; and *beta-actin*, forward 5′-ATC TGG CAC CAC ACC TTC TA-3′, reverse 5′-CGT CAT ACT CCT GCT TGC TGA TCC ACA TCT GC-3′. The experiments were performed three times each in triplicate.

### Conditioned medium

For conditioned medium, 2 × 10^4^ PrEC cells were seeded in a 35-mm dish containing optimal growth medium, and cultured with or without 100 ng ml^-1^ rFABP4, and the presence or absence of 30 μM BMS309403 for 24 hours. In addition, 1 × 10^5^ PCa cells were cultured in a 35-mm dish containing 1% FBS DMEM with or without 50 nM FABP4 siRNAs or 100 ng ml^-1^ rFABP4 for 24 hours. In some experiments, 2 × 10^4^ PrSC cells were seeded in the conditioned medium of PCa cells derived from the above experiments, and cultured with or without 30 μM BMS309403 for 24 hours. The conditioned medium of PrSC or PCa cells was then collected and stored at −80 °C until use.

### Cytokine analysis using cytometric bead array

Cytokine levels in the conditioned medium of PrSC, PCa cells and mouse serum were measured using a Cytometric Bead Array (CBA™, BD Biosciences, San Jose, CA, USA). This array simultaneously measures 6 proinflammatory cytokines, IL-8, IL-1β, IL-6, IL-10, TNFα, and IL-12p70. The procedure was carried out according to the manufacturer’s instructions. Briefly, 50 μl of chemokine capture bead mixture was incubated with 50 μl of each recombinant standard or sample and 50 μl PE-conjugated detection antibody for 2 hours at room temperature. The mixture was then washed to remove unbound PE detection reagent, and data acquisition was performed by FACSCalibur flow cytometry (BD Biosciences). Analysis was performed using CellQuest software (BD Biosciences). The experiments were performed three times each in triplicate.

### Human matrix metalloproteinase (MMP) antibody array

The RayBio Human MMP Antibody Array kit was purchased from RayBiotech (Norcross, GA, USA). This array consists of 10 different antibodies spotted in duplicate onto membranes for semi-quantitative detection. Experiments were performed according to the manufacturer’s instructions. Briefly, array membranes were incubated for 30 min in a blocking buffer and then incubated for 5 hours with 1.0 ml of the conditioned medium collected from PC-3 cells treated with or without FABP4 siRNAs for 24 hours. The membranes were washed, and incubated with biotinylated antibodies overnight at 4°C. Then, the membranes were washed and the sandwiched antigens were detected by incubation with a peroxidase-labeled streptavidin solution diluted to 1:1,000 for 2 hours. Proteins were detected by enhanced chemiluminescence, and signals were captured by the CCD camera system AE-9300 Ez-Capture MG (ATTO Instruments, Tokyo, Japan). The signal intensity in the array images was quantified and scored by Densitograph software (ATTO). The fold changes among samples were calculated analyzed using the RayBio Antibody Array Analysis Tool provided by RayBiotech. The experiments were performed twice each in triplicate.

### Cell proliferation assay

In total, 1 × 10^4^ cells were seeded in a 96-well plate and cultured in DMEM containing 5% FBS without antibiotics. The cells were treated with siRNA (50 nM), or rFABP4 (30 and 100 ng ml^-1^), and cultured for 24 hours. In some experiments, the cells were cultured with conditioned medium from PrSC treated with or without 100 ng ml^-1^ of rFABP4. Cell proliferation was assessed using a nonradioactive 3-(4,5-dimethylthiazol-2-yl)-2,5-diphenyltetrazolium bromide (MTT)-based cell proliferation assay kit (Roche, Basel, Switzerland). The experiments were performed in triplicate.

### Matrigel invasion assay

An *in vitro* invasion assay was performed in triplicate using Growth Factor Reduced BD BioCoat Matrigel Invasion Chambers (BD Biosciences) according to the manufacturer’s instructions. Briefly, 3 × 10^4^ cells were seeded in the upper chamber with conditioned medium from PrSC treated with or without 100ng ml^-1^ rFABP4, and the presence or absence of 10  μg ml^-1^ of IL-8 blocking antibody, IL-6 blocking antibody, control goat IgG, or control mouse IgG (R&D Systems, Minneapolis, MN, USA). In the siRNA experiments, cells were treated with 50 nM FABP4 siRNAs for 24 hours before being seeded in the chambers. Subsequently, 20% FBS DMEM was placed in the lower chamber, followed by incubation for 24 hours. In some experiments, 1 × 10^4^ PrSC were seeded in the lower chamber with optimal medium. Sera from mice were collected, clarified by filtration (SLGV004SL; Millipore, Billerica, MA, USA), and used for *ex vivo* cell invasion assays. Briefly, 3 × 10^4^ cells were seeded in the upper chamber with medium containing 5% FBS or 5% mouse serum with or without 10  μg ml^-1^ of IL-8 blocking antibody or 30 μM of BMS309403. DMEM with 20% FBS was placed in the lower chamber. Then, the non-invading cells in the upper chamber were removed and the membranes were stained with a Diff-Quik cell-staining kit (Sysmex, Kobe, Japan) to count the invading cells. The experiments were performed twice each in triplicate.

### Gelatin zymography

MMP2 and MMP9 enzymatic activities in the conditioned medium of PCa cells were assessed by a Gelatin zymography assay. Briefly, 3 × 10^5^ cells were seeded in a 6-well plate and treated with or without 50 nM of FABP4 siRNAs or 100 ng ml^-1^ of rFABP4 for 24 hours. In some experiments, the cells were previously treated with 10 μM of LY294002 or 1 μM of U0126 1 hour before rFABP4 administration. Subsequently, the conditioned medium was collected, and equal amounts of proteins (10 μg) were separated by sodium dodecyl sulfate-polyacrylamide gel electrophoresis on a gel containing 0.1% gelatin under non-reducing conditions. Next, the gel was washed in 2.5% Triton X-100 to remove sodium dodecyl sulfate and incubated in a development buffer for 20 hours at 37°C. The gelatinolytic activity was visualized after staining with 0.1% Coomassie Brilliant Blue, and the gel was destained in 50% methanol and 10% acetic acid. The bands were quantified using CS Analyzer (2.0) software (ATTO). The experiments were performed three times each in triplicate.

### Western blotting

Proteins were extracted from the cultured cells using Complete Lysis-M buffer (Roche, Switzerland). Equal amounts of protein (10 μg) were separated by sodium dodecyl sulfate-polyacrylamide gel electrophoresis, transferred to a polyvinylidene difluoride filter (ATTO) and hybridized with the following antibodies: anti-FABP4 (Abcam, Cambridge, UK), anti-α smooth muscle actin (αSMA, Abcam), anti-AKT, anti-phospho-AKT (P-AKT, Ser^473^), anti-ERK1/2, anti-phospho-ERK1/2 (P-ERK1/2, Thr202/Tyr204), or anti-beta-actin (Cell Signaling). The bands were quantified using CS Analyzer (2.0) software (ATTO). Western blots experiments were performed three times.

### FABP4 levels in the serum and conditioned medium

Human serum samples were obtained from 104 PCa patients and 40 healthy controls. The mean age and BMI levels of PCa patients and healthy controls were 69.0 ± 7.6 years and 24.1 ± 2.2 kg m^-2^, and 61.1 ± 14.8 years and 22.6 ± 3.1 kg m^-2^, respectively. The Institutional Review Board of the Akita University School of Medicine approved all experiments, and samples were obtained after written informed consent. The serum samples were obtained from the patients before they underwent anticancer therapy. Serum FABP4 levels were measured using a sandwich ELISA (R&D Systems), according to the manufacturer’s instructions. The concentration of human FABP4 in the conditioned medium of PCa cells was measured by human-specific ELISA. Mouse serum FABP4 levels were measured using a mouse FABP4 ELISA kit (CycLex Co., Ltd., Nagoya, Japan), according to the manufacturer’s instructions. The experiments were performed three times each in triplicate.

### Pathological histochemistry

Slides containing tissue samples from radical prostatectomy specimens were obtained from Akita University Hospital. The Institutional Review Board and Ethics Committee of the Akita University Graduate School of Medicine approved all experiments in this study and we obtained written informed consent for the use of all human samples related to this study. FABP4 rabbit polyclonal antibody (1:200, Abcam, Cambridge, UK) and α smooth muscle actin (1:200, αSMA, Abcam) were used as primary antibodies. Immunohistochemical staining was performed as described previously [11]. The proportion of positive αSMA stained stromal cells in the surrounding of cancer cells were evaluated, and the staining index was determined by the staining percentage score. The αSMA expression level was characterized as low-staining (≤10%) and high-staining (>10%) by the staining index. Slides of the tumor tissue from all mice underwent hematoxylin and eosin staining. The tissues and organ-specific metastasis in the peritoneum, intestine, stomach, liver, and diaphragm were assessed microscopically. Slides of mouse tumor tissues underwent immunohistochemistry to detect the expression of FABP4 (1:200, Abcam), αSMA (1:200, Abcam), smooth muscle actin (1:200, SMA, DAKO, Japan), Vimentin (1:200, Ventana Medical Systems, Tucson, AZ, USA), and Cytokeratin (1:100, AE1/AE3, DAKO). All evaluations and scoring of immunostaining were performed blinded by M.H. and H. N.

### Statistical analyses

All statistical analyses were performed using Microsoft Excel and SPSS ver12 software. All values are presented as the mean ± SD. Statistical significance was evaluated by an unpaired Student’s *t*-test or analysis of variance repeated measurement for comparison between two means in each experiment. Differences were considered statistically significant at *P* < 0.05.

## SUPPLEMENTARY MATERIALS FIGURES


