# Supplementary material for: Fatty acid binding protein 4 enhances prostate cancer progression by upregulating matrix metalloproteinases and stromal cell cytokine production
Source: Oncotarget. 2017 Dec 4;8(67):111780–94. doi: 10.18632/oncotarget.22908 (PMC5762359; doi:10.18632/oncotarget.22908)
Supplement: Supplementary file 1 [file oncotarget-08-111780-s001.pdf]

Fatty acid binding protein 4 enhances prostate cancer progression by upregulating matrix metalloproteinases and stromal cell cytokine production

SUPPLEMENTARY MATERIALS

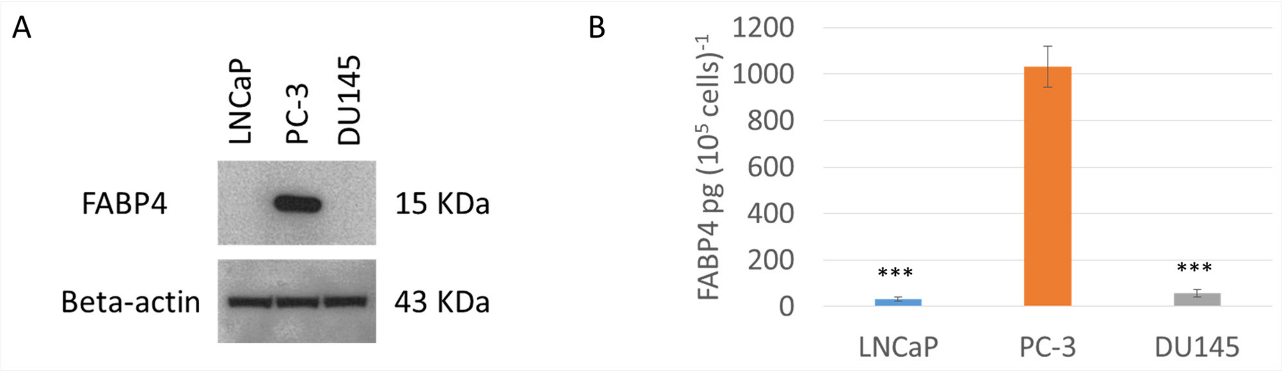

Supplementary Figure 1: FABP4 is highly expressed and secreted in PCa PC-3 cells. The 1 × 10^5 PCa LNCaP, PC-3, and DU145 cells were cultured in a 35-mm dish for 24 hours. Then, the conditioned medium was collected, and the cells were extracted. **(A)** An equal amount of protein from the cells was subjected to anti-FABP4 and anti-beta-actin antibodies. **(B)** FABP4 concentrations in the conditioned medium were measured by a human FABP4 ELISA kit. $^{***}P<0.001$.

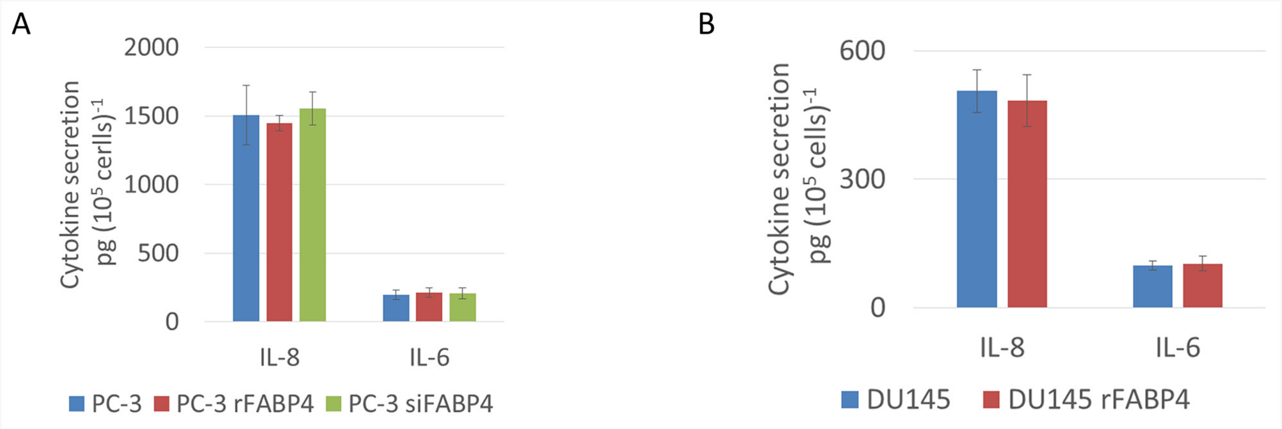

Supplementary Figure 2: FABP4 expression is not related to IL-8 and IL-6 secretion in PCa cells. The 1 × 10^5 PCa PC-3 **(A)** and DU145 **(B)** cells were seeded in a 35-mm dish, treated with 50 nM FABP4 siRNA-1 or 100 ng ml^{-1} rFABP4 and cultured for 24 hours. The conditioned medium was then collected and used for cytokine analysis using a Cytometric bead array kit.

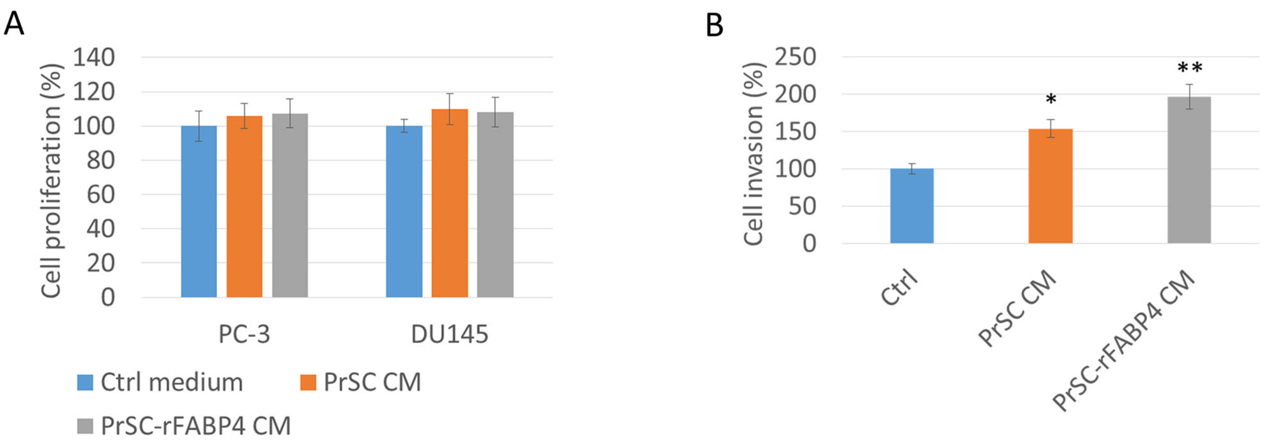

Supplementary Figure 3: PCa cell invasiveness but not cell proliferation is significantly increased by treatment of PrSCs with rFABP4. **(A)** The 1×10^4 PCa PC-3 and DU145 cells were seeded in a 96-well plate, and cultured in conditioned medium from PrSC cultured in the presence or absence of 100 ng ml^{-1} rFABP4 for 24 hours. The MTT assay was performed after 24 hours incubation, and cell viability was compared with untreated cells. **(B)** The histogram shows the invasion of DU145 cells stimulated in conditioned medium from PrSC cultured in the presence or absence of 100 ng ml^{-1} rFABP4 for 24 hours. All invading cells were counted and compared with untreated cells; $^{*}P < 0.05$ and $^{**}P<0.01$.

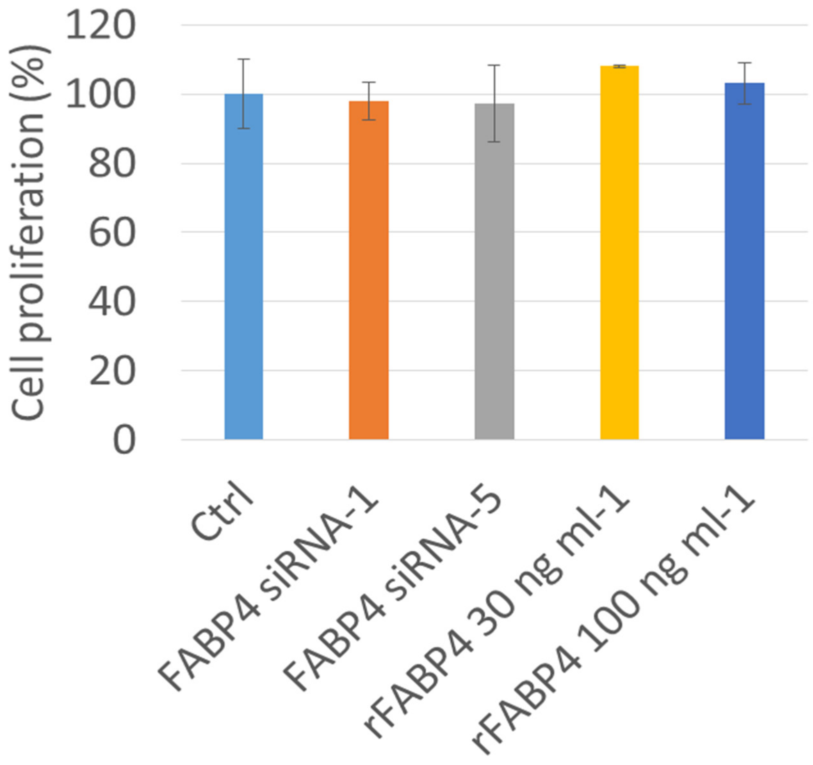

Supplementary Figure 4: FABP4 expression does not affect PCa PC-3 cell growth. The 1×10^4 PC-3 cells were seeded in a 96-well plate, and treated with 50 nM FABP4 siRNAs or 100 ng ml^{-1} rFABP4. The MTT assay was performed at 24 hours after incubation, and cell viability was compared with untreated cells.

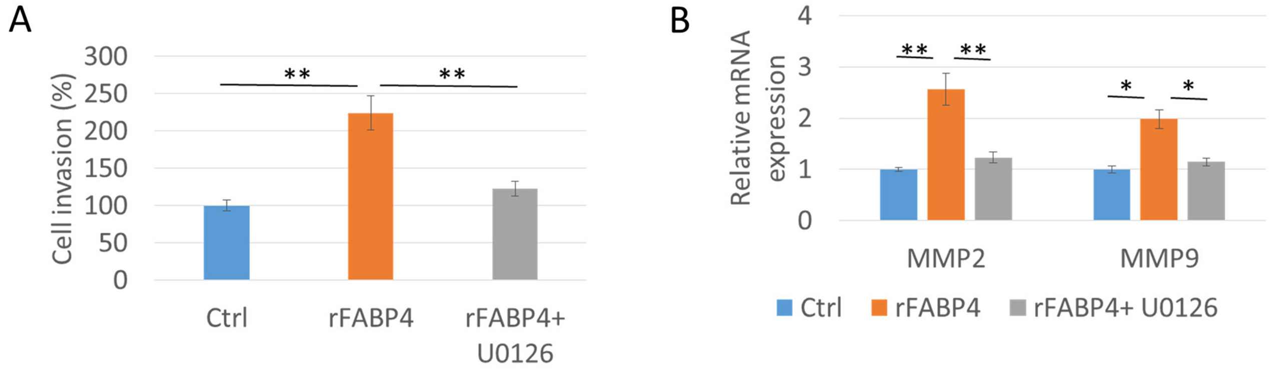

Supplementary Figure 5: FABP4 stimulates PCa DU145 cell invasiveness by upregulating MMP2 and MMP9 via ERK activation. **(A)** *In vitro* invasion assay. DU145 cells were treated with 100 ng ml^{-1} rFABP4 or 10 µM U0126 for 1 hour before treatment with rFABP4. After 24 hours, all invading cells were counted and compared with untreated cells; $^{**}p < 0.01$. **(B)** DU145 cells were treated with 100 ng ml^{-1} rFABP4 or 10 µM U0126 for 1 hour before treatment with rFABP4. After 24 hours, *MMP2* and *MMP9* mRNA levels were measured by quantitative RT-PCR, and compared with untreated cells. $^{*}P < 0.05$ and $^{**}p < 0.01$.

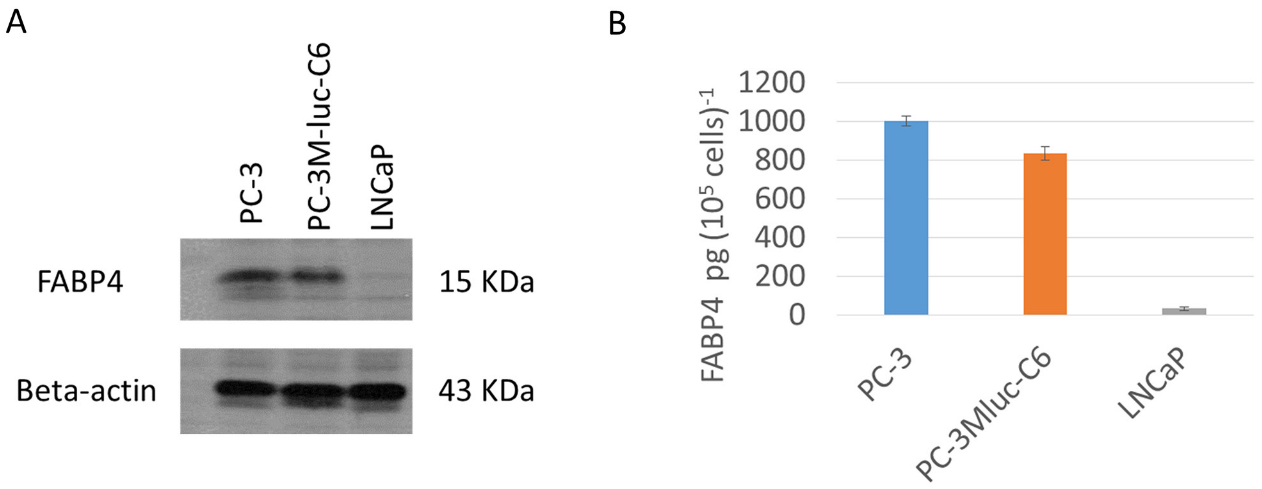

Supplementary Figure 6: FABP4 is highly expressed and secreted in PC-3M-luc-C6 cells and PC-3 cells. The 1×10^5 PCa PC-3, PC-3M-luc-C6 and LNCaP cells were cultured in a 35-mm dish for 24 hours. Then, the conditioned medium was collected, and the cells were extracted. **(A)** An equal amount of protein from the cells was subjected to anti-FABP4 and anti-beta-actin antibodies. **(B)** FABP4 concentrations in the conditioned medium were measured by a human FABP4 ELISA kit.

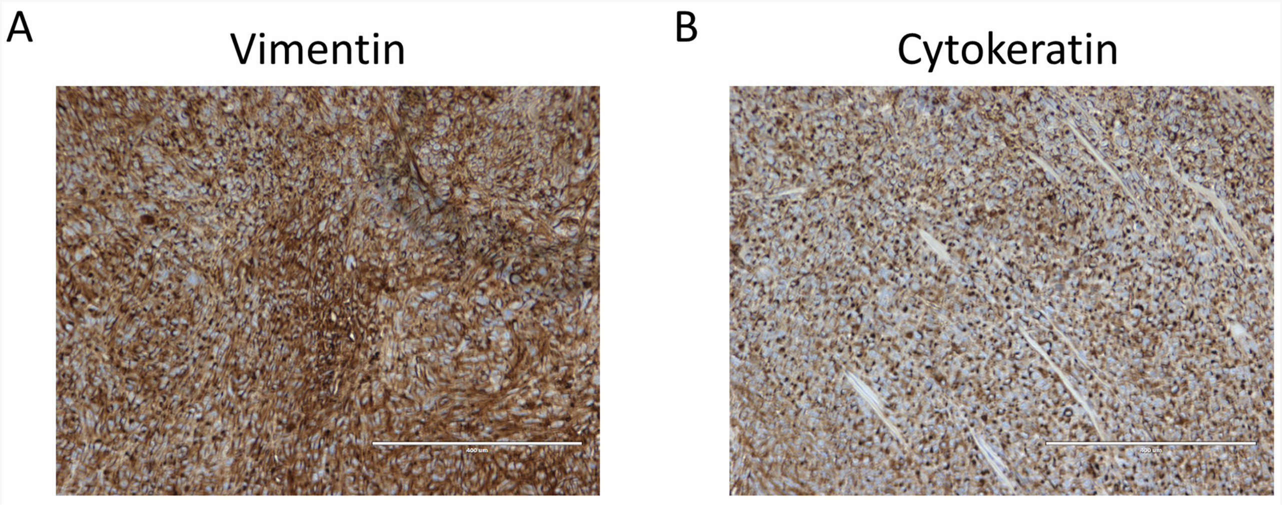

Supplementary Figure 7: Vimentin expression **(A)** and cytokeratin expression **(B)** in human PCa. Immunohistochemistry of mouse tumor tissues to detect the expression of vimentin and cytokeratin (AE1/AE3).

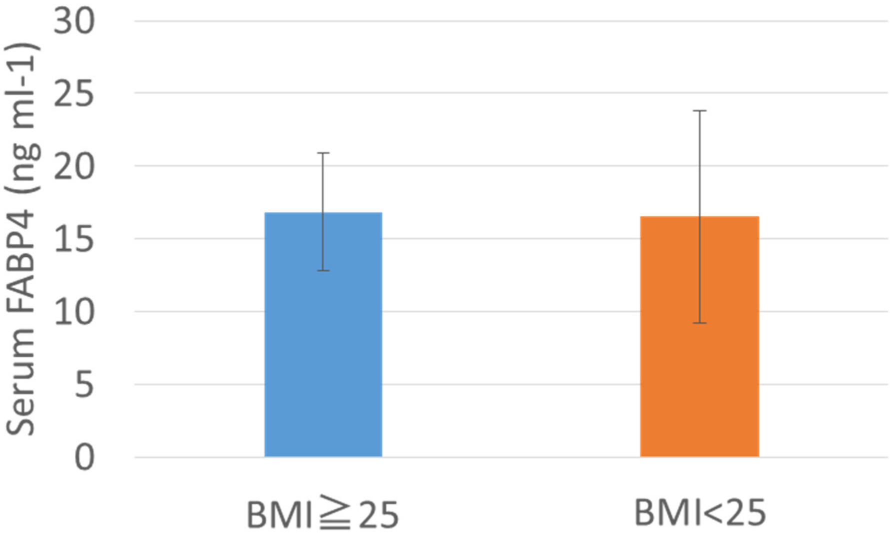

Supplementary Figure 8: The relationship between serum FABP4 levels and body mass index (BMI) in PCa. There were no statistical differences between the serum FABP4 level in PCa patients with a BMI < 25 (n=76) and with s BMI ≥ 25 (n=28) (16.5 ± 7.3 ng ml^{-1} vs 16.8 ± 4.0 ng ml^{-1}, P=0.471).
